# THE ROLE OF HEALTH IN RETIREMENT PLANNING TOWARDS EXTENDED WORKING LIVES

**DOI:** 10.1093/geroni/igae098.3903

**Published:** 2024-12-31

**Authors:** Rachel Crossdale, Nehle Penning

**Affiliations:** The University of Sheffield, UK, University of Sheffield, England, United Kingdom; TU Dortmund University, Dortmund, Nordrhein-Westfalen, Germany

## Abstract

Decades of research have shown that health is a key determinant of labour force participation in later life. As the ageing global population necessitates extended working lives (EWLs) for broader economic sustainability, the importance of the older population for the labour market is emphasised. Understanding the role of health and health inequality across the life course is crucial for impactful policy development to protect the health of the older workforce and encourage participation in EWLs. Using 100 interviews on risks and turning points in working life with older workers in Germany, Poland, Sweden, and the UK, this research explores the role of health as a barrier and facilitator of EWLs across the working life course. Drawing on policy and literature to contextualise our findings, this research reveals the role of health in retirement planning, including assumptions about health decline, impact on work, and the importance of partner’s health. Health as a ‘push and pull’ factor is also explored, with good health both fostering labour market engagement and encouraging early retirement, while physical and mental health can be both positively and negatively impacted by work. Inequalities by sector of employment, gender, and education level are also explored, with all four countries facing a ‘double bind’ whereby the most vulnerable must work longer for financial security but are limited by poor health. For future older populations to be healthier and more productive the current treatment-based model of health care needs to be challenged with preventative policy interventions throughout the life course.

